# *In vivo* Single Cell Optical Ablation of Brain Pericytes

**DOI:** 10.3389/fnins.2022.900761

**Published:** 2022-05-30

**Authors:** Cara D. Nielson, Andrée-Anne Berthiaume, Stephanie K. Bonney, Andy Y. Shih

**Affiliations:** ^1^Center for Developmental Biology and Regenerative Medicine, Seattle Children’s Research Institute, Seattle, WA, United States; ^2^Graduate Program in Neuroscience, University of Washington, Seattle, WA, United States; ^3^Department of Neuroscience, Medical University of South Carolina, Charleston, SC, United States; ^4^Department of Pediatrics, University of Washington, Seattle, WA, United States; ^5^Department of Bioengineering, University of Washington, Seattle, WA, United States

**Keywords:** capillary, blood flow, pericyte, blood-brain barrier, two-photon imaging

## Abstract

Pericytes have myriad functions in cerebrovascular regulation but remain understudied in the living brain. To dissect pericyte functions *in vivo*, prior studies have used genetic approaches to induce global pericyte loss in the rodent brain. However, this leads to complex outcomes, making it challenging to disentangle the physiological roles of pericytes from the pathophysiological effects of their depletion. Here, we describe a protocol to optically ablate individual pericytes of the mouse cerebral cortex *in vivo* for fine-scale studies of pericyte function. The strategy relies on two-photon microscopy and cranial window-implanted transgenic mice with mural cell-specific expression of fluorescent proteins. Single pericyte somata are precisely targeted with pulsed infrared laser light to induce selective pericyte death, but without overt blood-brain barrier leakage. Following pericyte ablation, the changes to the local capillary network and remaining pericytes can be examined longitudinally. The approach has been used to study pericyte roles in capillary flow regulation, and the structural remodeling of pericytes involved in restoration of endothelial coverage after pericyte loss.

## Introduction

Pericytes are mural cells that cover brain capillaries. They have ovoid cell bodies that protrude from the capillary wall and long processes that span along the longitudinal axis of the vessel. Thin-strand pericytes, which are the most common pericyte type in the capillary bed, extend their processes until they meet the terminal tip of neighboring pericyte processes, forming a chain-like network that contacts more than 90% of the capillary endothelium (Hartmann et al., 2015a; Underly et al., 2017; Grant et al., 2019). Pericytes are embedded in the vascular basement membrane, forming peg-and-socket connections with endothelial cells (Ornelas et al., 2021). This close apposition allows pericytes to serve many essential roles, including formation and maturation of blood vessels (Gerhardt and Betsholtz, 2003; von Tell et al., 2006), integrity of the blood-brain barrier (BBB) (Armulik et al., 2010; Daneman et al., 2010; Vazquez-Liebanas et al., 2022), and control of capillary tone and blood flow (Hall et al., 2014; Kisler et al., 2017; Berthiaume et al., 2018; Hartmann et al., 2021).

In Alzheimer’s disease (AD) and related dementias, pericytes are lost at an accelerated rate compared to normal aging. *Post-mortem* tissue analysis of patients with AD shows a ∼25–50% reduction in the number of pericytes compared to healthy controls (Sengillo et al., 2013; Miners et al., 2018). In the acute condition of focal ischemia, rapid pericyte death is seen, and aberrant contraction of surviving pericytes contributes to capillary constriction and a lasting hypoxic environment (Yemisci et al., 2009; Fernández-Klett et al., 2013; Hall et al., 2014). Traumatic brain injury also results in pericyte loss in the acute phase post-injury, followed by a later phase of pericyte detachment and migration from the vessel wall (Dore-Duffy et al., 2000; Zehendner et al., 2015). The effect of pericyte loss on vascular function and cerebral blood flow has remained poorly defined, given the complexity of these pathologies and the inability to resolve capillary level changes with *in vivo* clinical imaging.

The pathophysiological effects of pericyte loss have largely been studied using genetic pericyte ablation approaches. There are a variety of mouse models exhibiting congenital pericyte deficiency created by perturbation of signaling pathways required for pericyte recruitment during brain development. For example, mice with platelet-derived growth factor receptor-β (PDGFRβ) haploinsufficiency, and mice with a reduced retention of endothelium-produced platelet-derived growth factor-B, develop progressive pericyte loss, dilated and sparser capillaries, and marked BBB disruption (Lindblom et al., 2003; Armulik et al., 2010; Bell et al., 2010; Daneman et al., 2010). These pioneering studies were the first to identify pericyte roles in brain vascular function. However, congenital pericyte deficiency may not accurately represent the loss of pericytes that occurs with aging, or injury and disease in the adult brain. To this end, the extensive dilation of microvessels and reduction in capillary density that occurs with congenital pericyte loss is not observed in mouse models of adult onset pericyte loss (Vazquez-Liebanas et al., 2022).

Diphtheria toxin induced cell death is a strategy for more rapid, global loss of brain pericytes in adulthood. Two versions of the approach have been implemented with somewhat mixed outcomes. With one strategy, mice expressing human diphtheria toxin receptors in pericytes are injected with diphtheria toxin to induce cell death (Nikolakopoulou et al., 2019; Kisler et al., 2020). This results in progressive pericyte loss to 50–60% of basal levels and marked decrease in blood flow, BBB disruption and neurodegeneration. In a second approach, only diphtheria toxin is genetically expressed in mural cells, including pericytes. This approach also leads to marked loss of pericytes with widespread blood flow deficits and tissue hypoxia, but there is a surprising lack of BBB dysfunction (Park et al., 2017; Choe et al., 2022). This discrepancy may be due to off-target effects of the toxin provided systemically, though more studies are needed. Global pericyte loss produces complex pathophysiological effects, often making it difficult to discern pericyte roles in normal physiology.

Recently, 2Phatal (two-photon chemical apoptotic targeted ablation) was developed to induce single cell apoptosis *in vivo* (Hill et al., 2017). The method uses a femtosecond pulsed laser to excite cell-permeant DNA binding dye Hoechst 33342 within the nuclei of targeted cells. This leads to production of free radicals, which damages the irradiated cell and results in apoptotic cell death. Precise ablation of single pericytes of the murine brain has been demonstrated with 2Phatal. The cell death occurs over days and involves increases in Annexin V labeling consistent with apoptosis. However, this approach requires Hoechst 33342 delivery via topical application to the mouse cortex or via intravenous injection, which may have unforeseen effects on cellular function. Further, given that Hoechst 33342 labels the nuclei of many cell types, it is possible that observational imaging induces subthreshold and non-specific damage to cells during routine imaging.

Here, we describe optical ablation of single pericytes in mouse cerebral cortex during two-photon microscopy, without the need for an exogenous photosensitizer. This methodology relies on spatially restricted thermal injury to induce rapid loss of single pericytes, likely by necrosis, without overt disruption to the BBB. We discuss the resources required for the approach, step-by-step methods for ablation, longitudinal imaging post-ablation, advantages and limitations. Optical pericyte ablation *in vivo* has already been used to elucidate pericyte roles in maintenance of basal capillary tone, and pericyte remodeling to restore endothelial coverage following pericyte loss (Berthiaume et al., 2018; Hartmann et al., 2021).

## Materials and Equipment

The mice, reagents, consumables, and equipment necessary to perform optical pericyte ablations and image and quantify pericyte remodeling are listed in Table 1.

**TABLE 1:** Materials for single cell pericyte ablations *in vivo*.

Item	Supplier
**Mouse lines**	
Tg (Pdgfrb-Cre)*^35Vli^* (PDGFRβ-Cre) mice	Cuttler et al., 2011
B6.Cg-*Gt (ROSA)26Sor^tm14(CAG–tdTomato)Hze^*/J (Ai14) mice	JAX, 007914
**Reagents and consumables**	
Fluorescein isothiocyanate-dextran (FITC-Dextran 70 kDa)	Millipore Sigma, 46945
Phosphate buffered saline	Millipore Sigma, P3813
Isoflurane	Patterson Veterinary, 07-893-1389
Sodium chloride ophthalmic ointment	Akorn, NDC: 17478-622-35
U-100 BD ultra-fine™ short insulin syringes	VWR, BD328438
Cotton tipped applicators	ULINE, S-18991
**Equipment**	
Two-photon microscope with line-scanning capabilities	Any manufacturer
Digital power meter console	Thorlabs, PM100D
Thermal power sensor	Thorlabs, S350C
**Software**	
ImageJ/FIJI	https://imagej.nih.gov/ij/
SNT plugin for ImageJ/FIJI	https://imagej.net/plugins/snt/

*Materials and Equipment*

### Methods

#### Animals

PDGFRβ-Cre mice (Cuttler et al., 2011) were bred in-house with Ai14 reporter mice to create PDGFRβ-tdTomato mice (Hartmann et al., 2015b). These mice express tdTomato in capillary pericytes, but also in mural cells and perivascular fibroblasts of arterioles and venules (Bonney et al., 2021). In principle, pericyte ablation could be performed as described using any transgenic mouse line with bright fluorescent labeling of pericytes (Hartmann et al., 2015b). However, we have found that robust tdTomato expression through the Ai14 reporter provides the best results, due to clear and rapid loss of the fluorophore throughout the cell upon ablation.

#### Surgery

Chronic cranial windows (skull-removed, dura intact) were implanted over the sensorimotor cortex of PDGFRβ-tdTomato mice. The dual cover-glass “plug” method was utilized to ensure a stable imaging area over time. We direct the reader to detailed protocols for skull-removed cranial window techniques (Holtmaat et al., 2009). Briefly, anesthesia was induced with a cocktail consisting of fentanyl citrate (0.05 mg/kg), midazolam (5 mg/kg) and dexmedetomidine hydrochloride (0.5 mg/kg) (all Patter son Veterinary). Dexamethasone (20 μL; Patterson Veterinary) was given 3–6 h prior to surgery to reduce brain swelling during the craniotomy. Surgeries were performed under sterile conditions. During surgery, mice were placed on a feedback-regulated heat pad to maintain body temperature (FHC Inc.). Prior to craniotomy, a custom aluminum flange was fixed to the exposed skull with instant adhesive (Loctite Instant Adhesive 495). Under a stereo microscope (Olympus; SZX16), a ∼3 mm round craniotomy was created using a micro drill (OSADA; EXL-M40) and 0.5 mm burr (FST; 19007-05). Craniotomies were sealed with a glass coverslip consisting of a round 3 mm glass coverslip [Warner Instruments; 64-0720 (CS-3R)] glued to a round 4 mm coverslip [Warner Instruments; 64-0724 (CS-4R)] with UV-cured optical glue (Norland Products; 7110). The coverslip was positioned with the 3 mm side placed directly into the craniotomy, while the 4 mm coverslip laid on the skull surface. An instant adhesive (Loctite Instant Adhesive 495) was carefully dispensed along the edge of the 4 mm coverslip to secure it to the skull. Finally, Metabond dental cement was created by combining a single 0.15 cc scoop of Tooth Power (Patterson Dental; 553–3559) with 4 drops of Quick Base (Patterson Dental; 553–3492) and 1 drop of Catalyst (Patterson Dental; 553–3500) in a ceramic dish (Patterson Dental; 550–9906). The area around the cranial window, and any exposed areas of skull, was sealed with the dental cement using an applicator brush (Patterson Dental; 550–6787). This two-coverslip “plug” fits precisely into the craniotomy and helps to inhibit skull regrowth, preserving the optical clarity of the window. Following surgery, mice recovered for a minimum of 3 weeks before imaging.

#### Two-Photon Imaging

**FIGURE 1:**
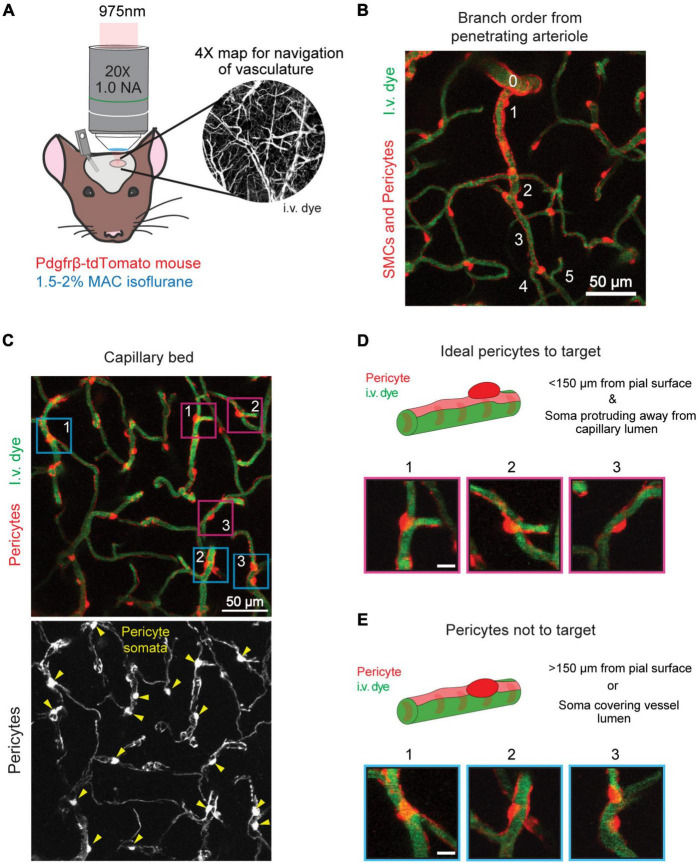
Selecting a pericyte for optical ablation. **(A)** *In vivo* two-photon imaging of an anesthetized PDGFRβ-tdTomato mouse with a 975 nm laser. Inset shows the 4X cranial window map used for navigation of the vasculature. **(B)** A penetrating arteriole (0) branching to form the arteriole-capillary transition zone (∼1–3 branch order), which further branches to form the capillary bed (≥4 branch orders). Thin-strand pericytes are found in the capillary bed. Pericytes are shown in red, and vessels are shown in green. **(C)** Capillary bed in the mouse cerebral cortex. Pericytes are labeled in red, and vessels are labeled in green. Lower panel shows pericytes in greyscale with yellow arrowheads indicating location of pericyte somata. Ideal pericytes to target are centered inside fuchsia boxes and pericytes that should not be targeted are centered in blue boxes. **(D)** Ideal pericytes to target for optical ablation. The schematic and following examples (1–3) contain a round pericyte soma that protrudes off the vessel wall. The fuchsia boxes in **(C)** correspond to each example. Scale bar = 10 μm. **(E)** Pericytes that should not be targeted for optical ablation. The schematic and following examples (1–3) contain a cell body that overlaps with an underlying vessel. The blue boxes in **(C)** correspond to each example. Pericytes are shown in red, and vessels are shown in green. Scale bar = 10 μm.

*In vivo* two-photon imaging was performed with a Bruker Investigator multiphoton microscope coupled to a Spectra-Physics Insight X3 laser source. Any two-photon imaging system may be used provided the appropriate excitation wavelengths are available from the laser, and line-scanning capabilities (preferably with user-defined scan orientations) can be implemented with the software. Green and red fluorescence emission was collected through 515/30 nm and 615/60 nm bandpass filters, respectively, and detected by gallium arsenide phosphide photomultiplier tubes (PMTs). The imaging system and laser were controlled with PrairieView 5.5 software. Mice were anesthetized with isoflurane in medical oxygen, using a plexiglass chamber (Braintree Scientific) for induction [4% minimum alveolar concentration (MAC)] and a custom nose cone and isoflurane vaporizer (Summit anesthesia) with an active scavenging vacuum system (Viking medical) during maintenance (1.5–2% MAC). During imaging, as during surgery, mice were placed on a feedback-regulated heat pad to maintain body temperature (FHC Inc.). The implanted aluminum flanges were affixed to a custom holder system to immobilize the animal’s head during imaging. To label the blood plasma, 70 kDa FITC dextran (Sigma-Aldrich), prepared at a concentration of 5% (w/v) in sterile phosphate buffered saline, was injected retro-orbitally. For each mouse, we obtain a wide-field map for navigation of the cerebrovasculature. This is achieved by collecting low-resolution images of the entire cranial window using a 4X (0.16 NA) objective (Olympus; UPlanSAPO) (Figure 1A). We then switched to a 20X (1.0 NA) water-immersion objective (Olympus; XLUMPLFLN) for observational imaging of pericyte (tdTomato) and vascular (FITC) structure, as well as for irradiation experiments. Observational imaging was performed at 975 nm (∼10–20 mW) to simultaneously excite tdTomato and FITC-dextran. Line scans for pericyte ablation and sham irradiation controls were performed at 725 nm (∼50 mW).

### Determine Pericyte Ablation Laser Power Settings

**Time:** 30 min

1. Turn on the multiphoton microscope and laser source.
2. Place the 20X objective onto the microscope.
3. Place the Thorlabs S350C Thermal Power Sensor under the objective and connect it to the Thorlabs PM100D Digital Power Meter Console.
4. Tune the laser to 725 nm.
5. Set the digital zoom in the imaging software to 15X to achieve a scan range similar to a line scan.
6. Determine the laser setting at which 50 mW of power is achieved at the output from the objective. This will be the setting used for pericyte ablations *in vivo*.

### Pericyte Ablation

**Time:** 1–2 h

**FIGURE 2:**
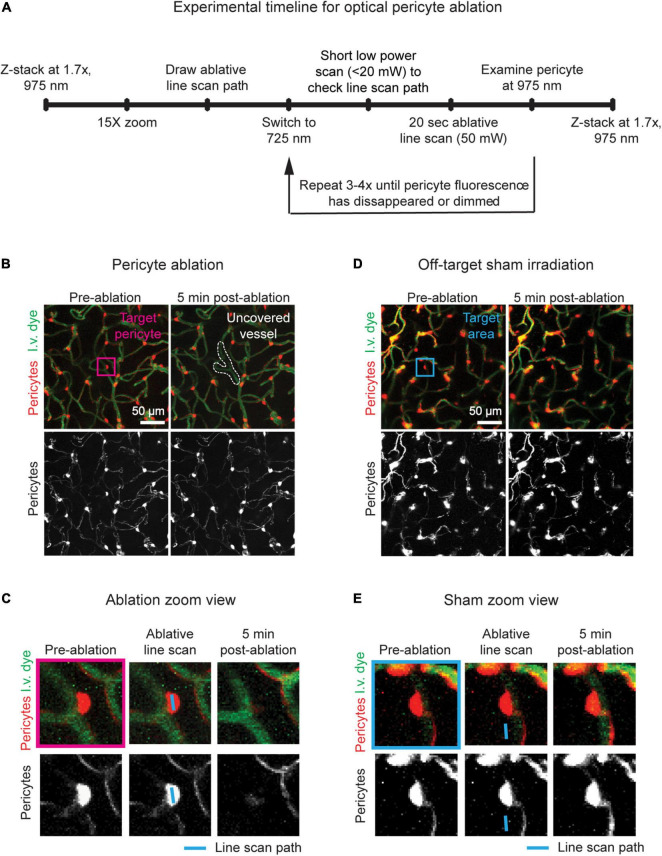
Timeline and examples of optical pericyte ablation and sham irradiation. **(A)** Procedural timeline to ablate single pericytes or perform sham irradiations. **(B)** Example of an on-target optical pericyte ablation. The area is shown before and 5 min after ablation. Pericytes are shown in red, and vessels are shown in green. The pericyte targeted for ablation is centered in the fuchsia box. Following ablation, the uncovered vessel area is outlined in white. **(C)** Zoomed in images of the pericyte of interest (fuchsia box in **C**) before ablation, with the line scan path overlayed, and 5 min after ablation. Scale bar = 10 μm. **(D)** Example of an off-target sham irradiation. The area is shown before and 5 min after irradiation. Pericytes are shown in red, and vessels are shown in green. The area targeted for sham irradiation is centered in the blue box. **(E)** Zoomed in images of the area of interest (blue box in **D**) before sham irradiation, with the line scan path overlayed, and 5 min after sham irradiation. Scale bar = 10 μm.

**FIGURE 3:**
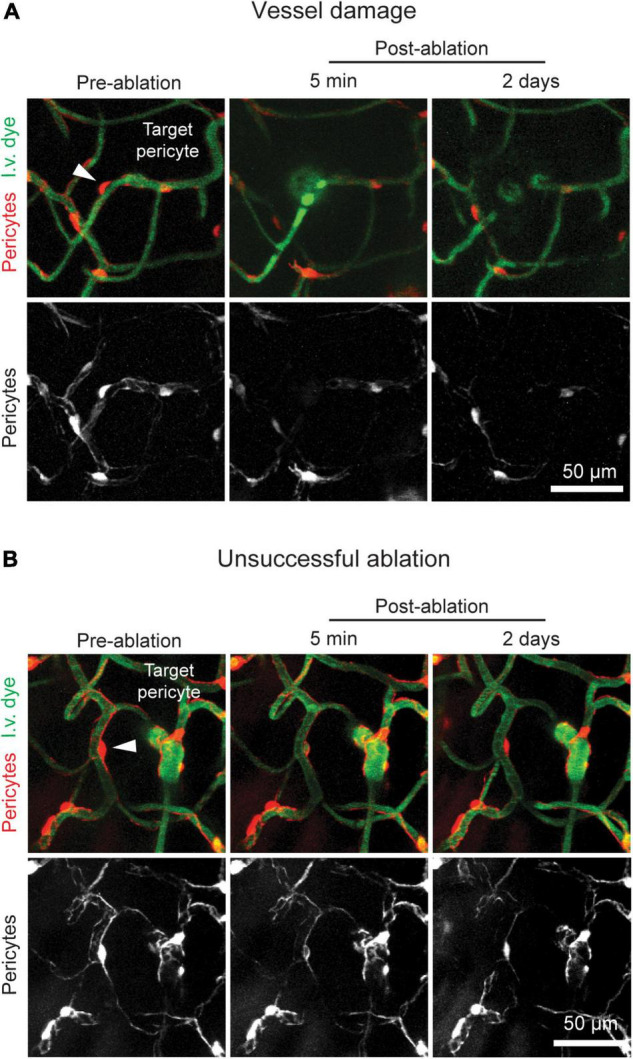
Potential pitfalls of optical pericyte ablations. **(A)** An example of a damaged vessel following pericyte ablation. **(B)** An example of a failed pericyte ablation. The area is shown prior to pericyte ablation and 5 min and 2 days post-ablation. Pericytes are shown in red, and vessels are shown in green. The arrowhead indicates the targeted pericyte.

1. Anesthetize PDGFRβ-tdTomato mouse with 4% MAC isoflurane in the induction chamber.
2. When the animal is deeply anesthetized (approximately 1 breath per second), place it on a heating pad at 37°C to maintain body temperature during imaging.
  (a) Place the animal’s snout into the nose cone.
  (b) Switch the anesthesia path from induction chamber to flow through the nose cone.
3. For anesthesia maintenance, reduce isoflurane to 1.5–2% MAC.
4. Affix the animals head to the custom flange holder.
5. Retro-orbitally inject 70 kDa FITC dextran to label blood plasma.
6. Apply ophthalmic ointment to the mouse’s eyes.
7. Clean off the cranial window with a cotton swab moistened with distilled water.
8. Align the cranial window under the 20X objective.
9. Place distilled water between the cranial window and the objective.
10. Direct the light path to the eyepieces and bring the cranial window into focus.
11. Direct the light path back to the PMTs and close the light shielding box for imaging.
12. Tune the imaging laser to 975 nm.
13. Begin scanning and utilize the 4X cranial window map of the vasculature (see *Two-Photon Imaging* methods) to locate an area on the cortical surface away from any major veins or arteries (Figure 1A).
14. Locate a thin-strand pericyte for ablation.
  (a) **NOTE:** Thin-strand pericytes are located in the capillary bed at the 4th branch order and beyond from a penetrating arteriole (Figures 1B,C).
  (b) **NOTE 2:** Ideal pericytes to target have an ovoid cell body that is protruding away from the capillary wall, which allows them to be targeted without hitting the vessel wall (Figures 1D,E). Ideal pericytes are less abundant than non-ideal pericytes.
  (c) **NOTE 3:** Pericytes with an ovoid cell body protruding away from the endothelium but nestled in the crux of a capillary junction are not ideal, but possible to ablate. The line scan would have to be sufficiently small to not damage the surrounding vessels (Figure 1E, example 3).
  (d) **NOTE 4:** Pericytes within the first 150 μm of the cortex are easiest to ablate.
15. Center the pericyte in the field of view Figure 2A.

16. Take a pre-ablation z-stack of the area from the cortical surface to a depth of approximately 200 μm (975 nm laser, 1.7X zoom, 1 μm z-steps, 512 × 512 pixels, 3.6 s dwell time, no averaging) (Figure 2B, left panels).
17. Ablate the pericyte.
  (a) Center the pericyte in the imaging window.
  (b) Lower the laser power and increase the zoom to 15X for precise observation of the soma Figure 2C, left panel.
  (c) Use the line scan tool to draw a line across the soma of the targeted pericyte (Figure 2C, middle panel).
  (d) Switch to the 725 nm laser.
    i. **Note 1:** Shorter laser wavelengths will cause more thermal injury compared to longer wavelengths that are used for routine *in vivo* multiphoton imaging, i.e., 800–1300 nm.
  (e) Set the line scan time to 20 s.
  (f) Perform a quick movie scan at low power (<20 mW) to ensure that the laser line scan path has not shifted.
  (g) Increase the laser power to ∼50 mW.
  (h) Begin the line scan.
  (i) After the line scan, switch back to 975 nm excitation, lower the power to normal levels for observational imaging (∼10–20 mW), and check if the pericyte has been ablated. If not, repeat c–h Figure 2C - right panel.
    i. **Note 1:** In general, pericytes will require 60–80 total seconds of 50 mW laser irradiation to be ablated (i.e., 3–4 rounds of 20 s line scans).
    ii. **Note 2:** Do not perform more than 4 iterations (80 total seconds of 50 mW laser irradiation). Higher irradiation times increase the risk of non-specific damage.
  (j) **Pause point:** Wait approximately 5 min.
  (k) If performing off-target sham irradiations, follow the above steps. However, draw the laser line scan path adjacent to the pericyte soma of interest, ensuring that the path does not overlap with the pericyte or the underlying vessel (Figures 2D,E). Perform 60-80 total seconds of 50 mW laser irradiation.
18. Use the pre-ablation image to best match the same field of view.
19. Take a post-ablation z-stack of the area from the cortical surface to a depth of approximately 200 μm (975 nm laser, 1.7X zoom, 1 μm z-steps, 512 × 512 pixels, 3.6 s dwell time, no averaging) (Figures 2B,D, right panels).
  (a) **Note:** If the pericyte retains bright fluorescence, and the ablative line scan has been performed less than 4 times, repeat step 17.
  (b) **Note 2:** If there is a breach in the capillary wall, exclude this ablation experiment from the study. Immediate leakage of i.v. dye (FITC dextran) may indicate damage to the vessel wall by the laser itself, warranting careful examination (Figure 3A).

20. Mark on the 4X cranial window map the location of the pericyte ablation. Using the orientation of large pial vessels in relation to the ablation site allows for easy navigation to the same spot during longitudinal imaging.
21. If ablating multiple pericytes, repeat steps 13–20.
  (a) **Note 1:** Mice are unlikely to experience negative behavioral outcomes from multiple ablations, given the highly focal nature of the manipulation.
  (b) **Note 2:** We suggest not keeping the animal under isoflurane anesthesia for longer than 2 h.
22. Remove the animal from isoflurane and allow it to recover in its home cage under a heating lamp.

### Longitudinal Imaging of Pericyte Remodeling

**Time:** ∼30 min per ablated pericyte

**FIGURE 4:**
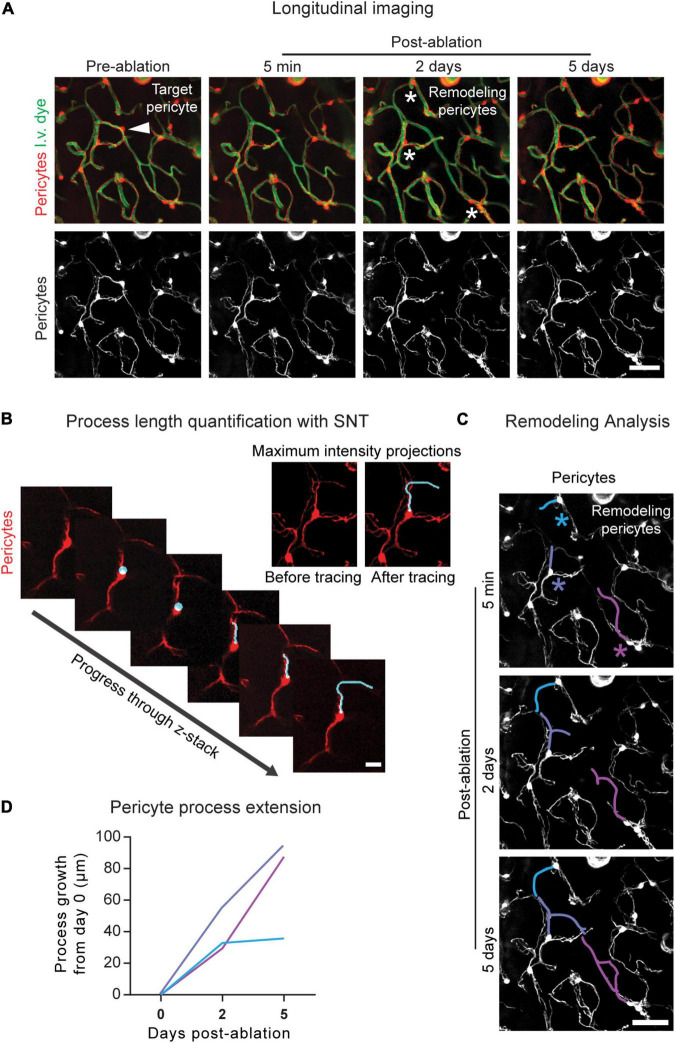
Longitudinal imaging and analysis of pericyte structural remodeling. **(A)** Example of longitudinal imaging following pericyte ablation. The area is shown prior to pericyte ablation and 5 min, 2 days, and 5 days post-ablation. In the upper left image, the arrowhead indicates the targeted pericyte. In the upper third image from the left, the asterisks indicate the remodeling pericytes. Pericytes are shown in red, and vessels are shown in green. Scale bar = 50 μm. **(B)** Pericyte length quantification in 3D using image z-stacks in ImageJ/FIJI. Using the SNT plugin, the process is traced from where it extends from the soma to its terminal tip. (Upper right) Maximum intensity projections of this pericyte process are shown prior to and following SNT analysis. Pericytes are shown in red. Scale bar = 15 μm. **(C)** Pericyte remodeling measured over time. Using the SNT plugin in ImageJ/FIJI the baseline lengths of the processes are measured and the growth of each pericyte process is measured at 2 and 5 days post-ablation using the post-ablation z-stacks. The colored asterisks correspond to the colored process at each imaging timepoint. Pericytes are shown in greyscale. Scale bar = 50 μm. **(D)** Quantification of pericyte remodeling. At each post-imaging timepoint, pericyte length is subtracted from pericyte length at baseline (day 0), and the resulting process growth in μm is plotted. Line color corresponds to the process color in **(C)**.

1. Repeat steps 1–12 in the *Pericyte Ablation* section.
2. Utilize the 4X cranial window map to navigate to the vascular area in which a pericyte was ablated.
3. Use the pre-ablation image to best match the same field of view.
4. Take a post-ablation z-stack of the area from the cortical surface to a depth of approximately 200 μm (975 nm laser, 1.7X zoom, 1 μm z-steps, 512 × 512 pixels, 3.6 s dwell time, no averaging) (Figures 2B,D, right panels).
  (a) **Note:** If the pericyte has regained fluorescence, exclude this ablation experiment from the study (Figure 3B).
5. Repeat steps 2–4 at all areas with ablated pericytes (Figure 4A).

6. Repeat at as many post-ablation time points as necessary.

### Pericyte Remodeling Analysis

**Time:** ∼1 h per ablated pericyte

Pericyte remodeling is quantified in ImageJ/FIJI using the SNT plugin, an open-source toolbox for semiautomated tracing, visualization, quantitative analyses, and modeling of neuronal morphology (Arshadi et al., 2021), built off the Simple Neurite Tracer program (Longair et al., 2011). We have adapted this tool for the tracing of pericyte processes and the quantification of pericyte process length. SNT can be downloaded at https://imagej.net/plugins/snt/.

1. Open the pre-ablation and all post-ablation z-stacks in ImageJ/FIJI.
2. From the “Analysis” drop-down menu, select “Set Scale,” and define the pixel to micron ratio of the images.
3. Examine the stacks and determine which neighboring processes are contributing to remodeling (Figure 4A).
4. Measure the baseline lengths and growth of the remodeling processes (Figures 4B,C).
  (a) Select the pre-ablation image to measure baseline process lengths or a post-ablation image to measure process growth.
  (b) Open the SNT plugin in ImageJ/FIJI.
  (c) In the SNT startup prompt:
    i. For “Image,” ensure the desired image stack is selected.
    ii. Leave “Reconstruction file” blank.
    iii. For “User interface,” select “Memory saving: Only XY view.”
    iv. For “Tracing channel,” select the tdTomato fluorescence channel number.
  (d) Turn off the green channel using the color channels tool.
  (e) Using the 3D image stack, trace each process.
    i. Click on the process from where it begins to extend from the soma.
    ii. Progress through the z-stack and click on a point further along the process. SNT will automatically trace a path between the two points.
    iii. Examine the path traced in SNT. If it is correct, select “Yes” under “Keep this new path segment?” If the path is incorrect, select “No” and try again.
      1. **Note:** Selecting points close to one another will increase tracing accuracy.
    iv. Continue tracing the process until you reach its terminus.
    v. Select “Finish.”
    vi. **Note:** When determining baseline process length (from soma to process terminus), examining the 5-min post-ablation stack may be helpful in determining where the process ends.
  (f) Measure the process.
    i. Select “Analysis,” then “Measure.”
    ii. In the “Metrics” pop-up box select “Length of primary branches (sum).”
  (g) Record the process length.
  (h) Repeat a–g for each process at each imaging timepoint.
5. Determine the growth of remodeling processes by subtracting the baseline process length from the new process length at each imaging timepoint (Figure 4D).

## Results

The targeted ablation of single pericytes via two-photon irradiation results in the total or near total loss of pericyte fluorescence in the soma and extending processes 5 min post-ablation (Figures 2B,C). If the pericyte retains high tdTomato signal, the ablative line scan should be repeated, but no more than 80 s of 50 mW laser irradiation should be applied to the cell. In some cases, the cell body and processes may appear dimmer, but not completely disappear until a later imaging timepoint. Fluorescence loss reveals the territory previously occupied by the ablated cell. The underlying capillary lumen can be visualized with the FITC dextran dye, and should be undisturbed immediately following pericyte ablation, with the exception of a dilation of the lumen in regions lacking pericyte coverage (Figures 2B,C). Off-target sham irradiations should typically produce no changes to the adjacent pericyte and its protruding processes. Occasionally, there may be a slight dimming in fluorescence of a pericyte neighboring an off-target sham irradiation, but the cell should not die (Figures 2D,E). This dimming typically resolves in the few days following the irradiation. If not, it is likely that the ablative line scan was targeted too close to the pericyte soma or its processes, and may need to be omitted from the study. This control procedure should be performed to verify that key results are due to pericyte loss rather than laser irradiation alone.

Successfully ablated pericytes do not regain fluorescence at any post-ablation imaging timepoint, indicating that pericyte death occurs through photo- and thermal toxicity. In some cases, a pericyte will regain fluorescence following ablation, likely due to temporary photobleaching or re-expression of tdTomato (Figure 3A). This can occur if ablations are performed with inadequate laser powers or irradiation times. In most cases, 50 mW of power targeted at a pericyte for 60–80 s is sufficient to induce cell ablation. Failed ablations may also occur if the targeted pericyte is too deep within the tissue. Cells in the upper 150 μm of the cerebral cortex are the easiest to ablate with the recommended settings. Past this cortical depth, higher laser powers for longer time periods may be required for pericyte ablation, which increases the risk of non-specific tissue damage.

If the underlying vessel or a nearby vessel is damaged by the line scan, immediate leakage of the dextran-conjugated dye from the vessel may be seen in the capillary bed. In severe cases, the vessel may rupture, producing a microbleed (Figure 3A). This outcome can occur for several reasons. If the animal is not in a deep enough anesthesia plane, it may move slightly during the ablative line scan leading to inaccurate targeting. To overcome this problem, ensure that the animal is adequately anesthetized, and that the anesthesia equipment is working properly. Vessel damage may also occur if the selected pericyte was not an ideal target. If the pericyte is not protruding sufficiently from the vessel wall or is close to another nearby vessel, the line scan path may hit the endothelium. Prior to ablation, ensure that pericytes are easy to target without risk of laser damage to the endothelium. This can be achieved by: (1) Toggling the tdTomato channel on and off after drawing the line scan path to check that the scan will not overlap or come very close to the capillary lumen and (2) checking that a capillary segment does not cross the pericyte immediately above or below the imaging plane. With successful ablations, we find that the focal loss of pericyte coverage is not sufficient to cause overt 70 kDa dye leakage in healthy adult mice, suggesting that barrier properties remain intact (Berthiaume et al., 2018).

Pericyte structural remodeling can be tracked longitudinally following ablation (Figure 4A). The neighboring pericyte processes that contribute to pericyte remodeling can be identified and their growth quantified over time. The SNT plugin in ImageJ/FIJI allows for the length determination of individual pericyte processes (Figures 4B,C). From this data, information on pericyte growth dynamics can be extracted. By subtracting the baseline process length from the process length at each post-ablation timepoint, the growth of each process over time can be calculated (Figure 4D). The maximum growth rate, average growth rate, and growth of non-contacted processes, i.e., processes that have not yet met the terminus of a neighboring pericyte process, can also be determined. Generally, ∼10–20 pericyte ablations per experimental group, performed across 4–6 mice, is sufficient for statistical power to be achieved (Berthiaume et al., 2018; Hartmann et al., 2021). These data allow us to understand the endogenous repair strategies mounted by the brain vasculature in the event of loss in pericyte coverage.

## Discussion

Single cell optical ablation of cortical pericytes can be used to examine the consequences of pericyte loss and pericyte remodeling in a precise and focal manner *in vivo*. This strategy allows for the ablation of single pericytes without the complex pathophysiological sequelae of global pericyte ablation. Further, this approach is relatively simple, rapidly induces pericyte loss, and decreases the likelihood of inducing non-specific damage with exogenous photosensitizers. Thus, the optical ablation approach complements other strategies for inducing global or focal pericyte loss, as researchers begin to unravel the functions of this still poorly understood cell type.

Single optical pericyte ablations can be unsuccessful either due to a damaged capillary or the resurgence of pericyte fluorescence. With experience, vessel damage and temporary photobleaching should rarely occur. However, new users might see a higher incidence of these occurrences as they hone their technique. In a worst-case scenario, they should not account for more than 30% of attempted ablations. These scenarios can be avoided with careful attention to the state of mouse anesthesia, choice of target pericyte, and ablative line scan settings. The main pitfall of this approach is the inability to ablate pericytes in deeper regions of the cortex and subcortex. However, the ability to rapidly ablate single cortical pericytes with high spatial and temporal precision, and with minimal mouse breeding and handling is an invaluable technique for brain vascular research.

Single optical pericyte ablations can be performed not only through chronic cranial windows, but through acute and thinned-skull cranial windows as well (data not shown) (Drew et al., 2010). Although in thinned-skull windows, the pericytes chosen for ablation will need to be even closer to the cortical surface, due to light scattering by the skull. Ablations can also be performed effectively in both adult and aged mice. As such, this strategy can be used to interrogate the effects of pericyte loss in the aging brain and can be coupled with mouse models of disease to investigate pericyte loss and pericyte remodeling in various conditions. Given that NeuroTrace 500/525 fluorescently labels pericytes *in vivo* (Damisah et al., 2017), future studies should test whether it is possible to ablate NeuroTrace 500/525-labeled pericytes, removing the need for transgenic expression of fluorescent proteins in pericytes.

Examining the consequences of pericyte loss and the mechanisms of pericyte remodeling in a focal manner is essential to closing the knowledge gaps in the physiological role of pericytes and the consequence of their loss in capillary networks. With this method, pericyte roles in capillary perfusion can be teased apart and strategies to promote pericyte coverage can be tested.

## Data Availability Statement

The original contributions presented in the study are included in the article/supplementary material, further inquiries can be directed to the corresponding author/s.

## Ethics Statement

The animal study was reviewed and approved by the Institutional Animal Care and Use Committee at the Seattle Children’s Research Institute.

## Author Contributions

CN collected data, performed analyses, and wrote the manuscript with feedback from AS. A-AB and SB substantially contributed to the development of the technique and facilitated data collection. All authors contributed to the article and approved the submitted version.

## Conflict of Interest

The authors declare that the research was conducted in the absence of any commercial or financial relationships that could be construed as a potential conflict of interest.

## Publisher’s Note

All claims expressed in this article are solely those of the authors and do not necessarily represent those of their affiliated organizations, or those of the publisher, the editors and the reviewers. Any product that may be evaluated in this article, or claim that may be made by its manufacturer, is not guaranteed or endorsed by the publisher.
