# Corrigendum: *In vivo* Single Cell Optical Ablation of Brain Pericytes

**DOI:** 10.3389/fnins.2022.974311

**Published:** 2022-07-12

**Authors:** Cara D. Nielson, Andrée-Anne Berthiaume, Stephanie K. Bonney, Andy Y. Shih

**Affiliations:** ^1^Center for Developmental Biology and Regenerative Medicine, Seattle Children's Research Institute, Seattle, WA, United States; ^2^Graduate Program in Neuroscience, University of Washington, Seattle, WA, United States; ^3^Department of Neuroscience, Medical University of South Carolina, Charleston, SC, United States; ^4^Department of Pediatrics, University of Washington, Seattle, WA, United States; ^5^Department of Bioengineering, University of Washington, Seattle, WA, United States

**Keywords:** capillary, blood flow, pericyte, blood-brain barrier, two-photon imaging

Andrée-Anne Berthiaume and Stephanie K. Bonney were not included as authors in the published article. The corrected Author Contributions Statement appears below.

CN collected data, performed analyses, and wrote the manuscript with feedback from AS. A-AB and SB substantially contributed to the development of the technique and facilitated data collection. All authors contributed to the article and approved the submitted version.

The authors apologize for this error and state that this does not change the scientific conclusions of the article in any way. The original article has been updated.

## Publisher's Note

All claims expressed in this article are solely those of the authors and do not necessarily represent those of their affiliated organizations, or those of the publisher, the editors and the reviewers. Any product that may be evaluated in this article, or claim that may be made by its manufacturer, is not guaranteed or endorsed by the publisher.

